# Cystitis

**Published:** 1892-03

**Authors:** C. E. Murphy

**Affiliations:** Atlanta, Ga.


### CYSTITIS.

BY C. E. MURPHEY, M. D.,
ATLANTA, GA.

Cystitis is one of the diseases that taxes the patience and often the skill of the ablest physicians and surgeons.

There are a great many different forms and grades, and as many causes, which have assumed as many names, but as they all are stepping stones to different stages, I will only divide in two stages, acute and chronic.

Thus a patient from gonorrhœa, stone in the bladder, or from careless use of instruments receives an injury to the bladder, causing acute cystitis. This may slowly drift into chronic cystitis or, if the inflammation extends still further, involving the deeper coat of the bladder, we have another form still more serious and a stage in which a great deal of trouble may be expected. The urine contains pus in greater or less quantity, and from the intensity of the inflammation portions of the bladder walls may slough; in this case the urine contains shreds of broken down tissue and has a gangrenous odor.

Examination of the urine may show it to be alkaline and in a short time afterwards, at another examination, it will be acid. When the urine is allowed to stand for some time, sediments separate, consisting of muco-pus, tinged with a great deal of blood and a considerable number of epithelial cells. As the case increases in severity, the pus increases in quantity, the urine becomes more turbid, fermentation occurs, the urine acquires a disagreeable ammoniacal smell, micrococci appear in great numbers, giving a cloudy appearance to the urine and from this stage it advances slowly until death ends the scene, unless relieved.

Acute cystitis, from whatever cause, presents the above line of symptoms.

The exciting causes of cystitis come from many sources. Gonorrhœa and stone, perhaps, are two of the most prominent ; but it does often arise from direct injury to the bladder, irritation of the urine altered by different drugs. Acid urine decomposes urea, forming carbonate of ammonium, introduction (by use of instruments) of germs in the bladder, injury to the nervous system, extension of inflammation from other organs or neighboring parts and, some claim, from masturbation and stricture of the urethra, etc.

Diagnosis is usually easy to make, as the symptoms are so prominent and the urine can readily be examined and its condition watched.

Hypertrophy of the bladder is a common accompaniment of cystitis. You can recognize the thickening of muscular coat, lessening the cavity of the bladder, by attempt to wash out the bladder.

The introduction of a small quantity of water brings on expulsive contractions and the thickened condition can be felt through the rectum or vagina.

Pure neuroses of the neck of the bladder, pyelitis, urethritis and prostatitis resemble, in many respects, cystitis, but by close examination they can be easily recognized. In the treatment of cystitis, the measure of the first and greatest importance is absolute rest. The rest should be in bed, with hips slightly raised, in order that pressure may be taken off the neck of the bladder. Constitutional treatment consists in regulating the character of the urine, so that it shall be unirritating to the diseased bladder.

To render the urine less irritating give alkaline diuretics, demulcent drinks, etc. Citrate of potash is one of the most valuable alkaline diuretics and is often advantageously combined with buchu, uva ursi, triticum repens.

Opium should be used to allay pain, lessen excitability and relieve spasmodic action. I usually prefer suppositories of opium and belladonna, but if the spasm alone is causing the pain belladonna alone will often relieve it, which is preferable. Poultices over the hypogastrium and perineum or hip-baths are useful. The bowels should be kept regular and free in order to secure free action of the portal circulation and prevent straining at stool. Free action of the skin and bowels relieves the taxed kidneys and bladder, giving them less to do. Saline purgatives are better suited for this purpose. Sulphate of magnesia or a glass of a laxative mineral water given before breakfast usually acts nicely. Digestion should be watched with care, in fact, I have found cases that could not be relieved until the state of the digestion was improved.

The diet is an important factor that should not be overlooked; irritating articles of food should not be allowed, spirits, alcohol in all forms must not be allowed; coffee and tea should not be allowed. Nothing should be taken that disagrees in the least with digestion.

In a mild case of cystitis, I do not adhere strictly to all of the rules laid down here, but let the patient continue at business if desired, and by proper care and treatment he may be relieved in a short time. For example, the urine is too acid or too alkaline, acts sometime like a foreign body; it irritates and the bladder will make efforts to expel it. Deposits of any urinary solids in the viscus are likely to produce an irritable condition. Such cases being of a mild form can readily be relieved if not allowed to stand too long.

In advanced stages of cystitis local treatment can be employed advantageously, by washing out the bladder with medicated injections, if properly employed. The instrument I use for this purpose was devised by Dr. Skene, of New York; it is simple, but gives entire satisfaction. I use a soft rubber catheter, attached to a piece of rubber tubing by means of a small glass tube, thus making about two feet in all; to the end of the tube attach a small glass funnel and the instrument is complete, ready for use. The catheter should be surgically clean, soft and flexible. The whole instrument must be thoroughly cleansed with a solution of carbolic acid. The catheter should be introduced with great care, to prevent any injury to the inflamed organ. Instillation should be made very slowly, in order not to distend the bladder rapidly; ascertain the quantity of fluid the bladder will hold with comfort, and only use that amount, as overdistention is liable to do harm. This amount of fluid can be used several times during one washing. Introduce the catheter in the bladder, lower the funnel, permitting the urine to escape. Still leaving your catheter in place proceed to wash the bladder out by pouring the solution in the funnel, elevate it, allowing the contents to flow in slowly. The flow can be regulated according to the height the funnel is raised. It is very important not to let the air enter the bladder and this can be prevented by grasping the rubber tubing near the funnel, immediately after the flow has ceased until the funnel is filled for use again. An admirable bladder irrigation is: boracic acid solution, two per cent.; borax, or table salt, in a three or four per cent. solution. Chlorate of potash is sometimes used with advantage in a two per cent. solution, or listerine one drachm to two ounces of warm water. After irrigating the bladder well the application of astringents and alteratives are most beneficial. I have used nitrate of silver, sulphate of zinc, acetate of lead, and infusion of hydrastis canadensis with marked benefit.

Cystotomy is rapidly growing in favor, and is claimed to relieve patients after every other means has failed, by many surgeons. So great is the faith of some in cystotomy that they operate in all cases of chronic cystitis, but I am inclined to think it should be the last resort.

I will give you the report of the following cases which occurred in my practice, which will perhaps be of interest to some of your readers:

Mrs. L., age twenty-seven, married, general health and appearance good. I called to see her May 29, 1890. She had been confined to her bed for ten days, giving the following history: For several days she had had a burning sensation, and a frequent desire to make water; her urination becoming more frequent and painful, until she resorted to the use of laudanum to relieve pain. Had no appetite and bowels was constipated.

Upon examination of the urine I found it full of mucus, number of epithelial cells, and muco-pus tinged with blood. The urine was alkaline, diminished in quantity, dark in color, and ammonia was also present. I gave her the following mixture:

℞. Potassium citrate, ʒiv.

Fluid ext. triticum repens,

Tr. hyoscyam., aa ℥j.

Fluid ext. of buchu, ℥ss.

M. Water, q. s. to make ℥iij.

Sig.—Teaspoonful in water every four hours.

℞. Ext. belladonna,
Sulph. morphine, aa grs. v.
M. Make suppositories x.

Sig.—Introduce one into anus when in pain.

Also ordered saline laxative to be taken when necessary to keep the bowels open. Ordered for diet soft eggs, soup, milk and beef essence.

May 31. I found my patient suffering less pain in urinating, bowels had not acted, and could not retain her food. I ordered a glycerine enema, and gave her the following prescription, to be taken at meal-time:

℞. Lactopeptine, ʒiv.

Subnitrate bismuth, ʒij.

Tr. gentian comp., ℥ij.

Aquæ pep., q. s. ad. ℥iv.

Sig.—Teaspoonful at meal-time in water.

June 2. I found my patient in great pain, had no appetite, temperature was 102°, and she was extremely nervous. I gave her a half grain of morphine hypodermically, and ordered water to wash out the bladder. I used two per cent. boracic acid solution. After irrigating the bladder I used one grain nitrate of silver to the ounce of warm water. It gave some pain, but she felt great relief afterwards. I kept up the pepsin solution, and gave the following directions: Twenty grains of boracic acid to be taken in decoction of buchu every four hours; ten grains pil. hydrarg. and one grain of ipecac to be taken at bed-time.

June 3. Patient much improved, bowels moved off well and much less pain in urinating. I kept up the twenty grains of boracic acid, and would wash out the bladder as above stated every second day. My patient improved steadily, and in three weeks was discharged cured.

November, 1890. This case was turned over to me by Dr. Todd. The patient had been in bed for eleven months, and had the attention of a physician during that time. The family finally called in Dr. Todd. The doctor stated to the husband that he could not give the necessary attention she needed and referred him to me, and at the same time requested me to call at his office. Dr. Todd's diagnosis was gonorrhœal cystitis, and after a conversation with closed doors the husband acknowledged that he had gonorrhœa about the time she contracted this trouble. Therefore the doctor's diagnosis was correct. So that night I called to see her. I heard her pitiful groans before entering the room. There was painful and frequent desire to urinate, with vesical tenesmus; the perspiration had a urinous odor; the unhealthy condition of the skin had all signs of malnutrition; temperature 103°. The patient had been fed on opium for some time. Digestion bad and bowels constipated. The vagina was swollen and extremely tender, and there were several large bed sores on the body.

On examination of the urine I found a large amount of pus corpuscles, organic debris, blood, mucus, crystals of uric acid and urate of ammonia. Urine was acid. I gave her the following prescription:

℞. Tr. nux vom., ʒiij.

Tr. mur. ferri, ℥j.

Tr. cinchonæ co., ℥ij.

M. Glycerinæ, q. s. ad. ℥iv.

Sig.—Teaspoonful in water three times a day.

℞. Ext. belladonna, gr. vj.
Acet. morphiæ, gr. viij.
M. Ft. suppositories, xij.

Sig.—One when necessary to relieve pain.

I also ordered her abdomen to be poulticed with a light, hot bran poultice. Used enema to move bowels. For food I suggested soft eggs, beef essence and sweet milk and lacto-peptine and subnitrate of bismuth, to be given after food.

I attempted to wash out her bladder, but the pain was so great postponed it for a day or two. Her bladder only held about half ounce of water without great pain. I moved her bowels with calomel, soda and ipecac.

4th day. Chloroformed her and irrigated her bladder with a solution of boracic acid, not using over an ounce at a time. Afterwards I used nitrate of silver, one grain to the ounce of water, with half a drachm of F. E. hydrastis canadensis.

I repeated this treatment every third day. I ordered "santal miday" one bottle, and directed the nurse to give to her three times a day.

I persisted in this treatment and my patient gradually commenced improving. After three weeks could wash out the bladder without the use of chloroform. Her health and digestion generally improved. I also gave her boracic acid and buchu from time to time.

At this stage I reduced the opium each day, but this was a task. After four week's hard labor I left off the opium entirely. In twelve weeks she was entirely well and doing her housework.

I have several cases I desire to report but will postpone.

